# Buprenorphine reduces methamphetamine intake and drug seeking behavior *via* activating nociceptin/orphanin FQ peptide receptor in rats

**DOI:** 10.3389/fpsyt.2022.983595

**Published:** 2022-10-06

**Authors:** Fangmin Wang, Wenwen Shen, Yujia Cai, Xin Zhang, Han Du, Miaojun Lai, Huifen Liu, Evelyne Kohli, Wenhua Zhou

**Affiliations:** ^1^Zhejiang Provincial Key Lab of Addiction, Ningbo Kangning Hospital, School of Medicine, Ningbo University, Ningbo, China; ^2^UMR INSERM/uB/AGROSUP 1231, Team 3 HSP-Pathies, Labellisée Ligue Nationale Contre Le, Cancer and Laboratoire d’Excellence LipSTIC, Dijon, France; ^3^UFR des Sciences de Santé, Université de Bourgogne, Dijon, France

**Keywords:** buprenorphine, nociceptin/orphanin FQ peptide, substance use disorder, opioid receptor, methamphetamine

## Abstract

Buprenorphine, which has been approved for the treatment of opioid dependence, reduces cocaine consumption by co-activating μ-opioid receptors and nociceptin/orphanin FQ peptide (NOP) receptors. However, the role of buprenorphine in methamphetamine (METH) reinforcement and drug-seeking behavior remains unclear. This study investigated the effects of buprenorphine on METH self-administration and reinstatement of METH-seeking behavior in rats. We found that buprenorphine pretreatment had an inhibitory effect on METH self-administration behavior, and that buprenorphine at a dose of 0.3 mg/kg could inhibit motivation to respond for METH. Pretreatment with the NOP receptor antagonist thienorphine (0.5 mg/kg) or SB-612111 (1 mg/kg) could reverse the inhibitory effect of buprenorphine (0.1 mg/kg) on the METH self-administration. Moreover, treatment with buprenorphine (0.1 mg/kg and 0.3 mg/kg) significantly reduced the drug-seeking behavior induced by context or by METH priming but failed to reduce the drug-seeking behavior induced by conditional cues. Additionally, the NOP receptor antagonist SB-612111 reversed the inhibitory action of buprenorphine on the drug-seeking behavior induced by METH priming. The results demonstrated that buprenorphine reduced either METH intake or the drug-seeking behavior by activating NOP receptors, providing empirical evidence for the clinical use of buprenorphine in the treatment of METH relapse and addiction.

## Introduction

Methamphetamine (METH) is one of the most commonly used illegal drugs worldwide. According to recent estimates, approximately 35 million people worldwide use amphetamine-type stimulants, and the number of abusers continues to rise (1, 2). The National Drug Abuse Monitoring Annual Report (2016) reported that synthetic drug abusers accounted for 54.8% of the total drug abusers in China, with METH abusers alone accounting for 87.4% of all synthetic drug abusers (3). METH use not only causes damage to the physical and mental health but also leads to a series of socio-economic and judicial problems. To date, few medicines have had their effectiveness in treating METH use disorders or preventing relapse among METH users demonstrated with strong evidence (4). As a partial μ-opioid receptor (MOP) agonist, buprenorphine has been approved for the treatment of opioid dependence (5–8). In recent years, the use of buprenorphine has emerged in the treatment of cocaine addiction, even though only high doses of buprenorphine may have noticeable effects in suppressing the desire for cocaine (9) and reducing concomitant opiate and cocaine use (10). Sporadic clinical observations have suggested that buprenorphine also has an effect on METH use disorders. For example, one clinical trial has shown that 16 weeks of daily buprenorphine induce greater reductions in METH craving in 40 participants (11). Another clinical observation supports the efficacy and safety of buprenorphine as a short-term treatment for METH craving (12, 13).

In preclinical studies, buprenorphine effectively inhibited cocaine self-administration (14). Studies have shown that buprenorphine reduces cocaine intake and enhances dopamine release induced by cocaine (15), reduces cocaine-seeking behavior during extinction following acute cocaine priming injections (16), and blocks cocaine sensitization by increasing basal levels of glutamate expression in the nucleus accumbens (NAc) (17). Moreover, high-dose buprenorphine extended extracellular DA outflow in the caudate nucleus for 190 min, whereas low-dose buprenorphine reduced DA release. Both doses attenuated METH-induced DA peak effects (18). In addition to its classical MOP, delta opioid receptor (DOP), and kappa opioid receptor (KOP) bindings, buprenorphine also acts as an agonist and/or partial stimulator for the nociceptin/orphanin FQ (N/OFQ) peptide (NOP) receptor (19–21).

The NOP receptor is a G protein-coupled receptor, originally classified as belonging to the opioid receptor family (22). However, the endogenous ligands of other opioid receptors, including MOP, KOP, and DOP receptors, have little affinity for NOP receptor (23). N/OFQ and its NOP receptor are widely distributed in brain regions such as the ventral tegmental area (VTA) and the NAc, where both are largely co-expressed and may be involved in the control of drug dependence (24). Studies have found that endogenous N/OFQ activates NOP receptors to reduce the expression of cocaine (25, 26) or METH-conditioned place preference (CPP) (27). Intracranial injection of N/OFQ inhibits cocaine-induced DA release in the NAc, blocks cocaine-induced motor sensitization by activating NOP receptors (28), and attenuates METH-induced acute reward response (29) and METH withdrawal responses (30). Recently, evidence has shown that, through the co-activation of MOP and NOP receptors, buprenorphine is essential in reducing cocaine intake (14). Up to date, whether buprenorphine inhibit the METH self-administration and drug seeking behavior is still unclear.

Here we hypothesized that buprenorphine may exert an inhibitory effect on METH self-administration and cravings through its agonistic effects on NOP receptor. First, we observed systematically the effects of buprenorphine on METH self-administration behavior and motivation for METH. To elucidate this NOP receptor mechanism, we performed intensive pharmacological studies using NOP receptor antagonist thienorphine and SB-612111. Thienorphine, a novel analog of buprenorphine, acts as an antagonist at NOP receptor and an agonist at DOP, KOP and partial agonist at MOP (31). We could compare the pharmacology of buprenorphine and thienorphine in assay for METH reinforcement. We further observed the effects of buprenorphine on drug-seeking behavior induced by context after withdrawal, and on reinstatement of drug seeking behaviors induced by conditioned cues or METH priming in self-administered rats. Moreover, SB-612111, a selective NOP receptor antagonist (32), was used to determine whether buprenorphine mediates METH reinforcement and relapse through NOP receptor.

## Materials and methods

### Animals

Male Sprague–Dawley rats (*n* = 86) provided by the Experimental Animal Center of Zhejiang Province and weighing 280–300 g was used in the present study. The rats were housed in an airy and clean animal room under a 12-h light/12-h dark cycle (lights switched on at 8 a.m. and switched off at 8 p.m.) with constant temperature (22–24°C) and constant humidity (50–70%). Food and water were provided *ad libitum* in the home cage for all rats, but food for sucrose reinforcement rats at the beginning train was restricted. The experimental environment strictly complied with the regulations on the management of laboratory animals in China. The experimental procedures were approved by the Ethics Committee of the Laboratory Animal Use and Care of Ningbo University. All animal experiments were performed in accordance with the National Institutes of Health (NIH) Guide for the Care and Use of Laboratory Animals (8th Edition).

### Drugs

Methamphetamine was obtained from the Drug Intelligence and Forensic Center of the Ministry of Public Security (Beijing, China) and was dissolved in 0.9% sterile saline. Buprenorphine Hydrochloride Injection was purchased from TIPR Pharmaceutical Co., Ltd. (Tianjin, China). Thienorphine (*N*-cyclopropylmethyl-7α-[(*R*)-1-hydroxy-1-methyl-3-(thien-3-yl)-propyl]-6,14-endo-ethanotetrahydronororipavine) was obtained from the Beijing Institute of Pharmacology (Beijing, China) and dissolved in 3% dimethyl sulfoxide (DMSO) and diluted in 0.9% sterile saline to a final concentration of 1% DMSO. SB-612111 was purchased from Sigma-Aldrich (St Louis, MO, USA), dissolved in 3% DMSO, and diluted in 0.9% sterile saline to a final concentration of 1% DMSO. Control animals received the same amount of 0.9% sterile saline or vehicle (1% dimethyl sulfoxide). Sucrose pellets were purchased from BioServe (Frenchtown, NJ).

### Intravenous catheter surgery

All surgical procedures were performed with the animals under sodium pentobarbital anesthesia (50 mg/kg, i.p.) and the analgesic carprofen (5 mg/kg, s.c.) was given following surgery for two days. Rats were surgically implanted with a chronic intravenous indwelling catheter (33). The catheters were flushed daily with a 0.2 ml saline–heparin solution (25 U/ml heparin) to maintain catheter patency. To prevent infection, the rats were treated post-surgically with penicillin B (30 mg/kg, intramuscularly) every day. The animals were allowed to recover for at least 7 days. From the second week of training, catheter patency was tested by injecting 0.1 mL (10 mg/mL) of propofol through the catheter for sedation.

### Methamphetamine self-administration

Rats were trained to self-administer METH in operant chambers equipped with two nose-poke ports (ENV-114 M, Med Associates, Lafayette, IN, USA). The training consisted in daily 4-h sessions for 10 consecutive days under a fixed-ratio 1 schedule of reinforcement, as previously described (34, 35). Rats received a single METH infusion (0.05 mg/kg) following an active nose poke. Each infusion was paired with a 5-s illumination of light in combination with the noise of the infusion pump; together, these stimuli served as a discrete conditioned cue paired with the drug infusion. Following the infusion, a time-out period was imposed for 20 s, during which the response was recorded but produced no programmed consequences. Responding to the inactive nose-poke port had no programmed consequences. The rats were returned to their individual housing cages shortly after the session. Similar to a previous report (36), the rats exhibited reliable METH self-administration when an acquisition criterion required that the subjects’ active nose pokes varied by less than 10% over the course of three consecutive maintenance days. The apparatus was controlled using an IBM-compatible PC running a program written in Pascal (Borland Delphi 6.0). After the rats acquired the METH self-administration behavior for 10 days under the FR1 schedule, they were randomly assigned to five groups (n = 7 in each group) and injected with vehicle(saline), 0.01, 0.03, 0.1 or 0.3 mg/kg buprenorphine (s.c.) 15 min before the testing session.

To investigate the pharmacological mechanism by which buprenorphine inhibits METH reinforcement, we tested the two NOP antagonists in the present experiment. The SB-612111 concentration used for this study was chosen based on an effective *in vivo* dose at 1 mg/kg (32), and thienorphine concentration used at dose of 0.5 mg/kg based on its antinociceptive effect ED 50 value of 0.25 mg/kg (37). The rats were randomly assigned to six groups (*n* = 7 in each group) and injected with vehicle (saline plus 1% DMSO, s.c.), buprenorphine treated group (1% DMSO plus 0.1 mg/kg buprenorphine, s.c.), thienorphine treated group (0.5 mg/kg thienorphine plus saline, s.c.), SB-612111 treated group (1 mg/kg SB-612111 plus saline, s.c.) or another two groups with an injection of thienorphine (0.5 mg/kg, s.c.) or SB-612111 (1 mg/kg, s.c.) and 10 min later they received buprenorphine(0.1mg/kg, s.c.) administration. Testing of self-administration occurred at 15 min after the final drug injection.

### Motivation to respond for methamphetamine

The METH motivation was measured by using progressive ratio (PR) schedule, a task that directly measures the breakpoint at which an animal is unwilling to further work for reward. The PR reinforcement schedule required animals to progressively increase nose poking for each successive reward in the following series within a self-administration session. There was a timeout of 20 s following the infusion in the PR schedule. The progression of response requirements was calculated using the following equation: Response ratio = (5 × e (0.2 × infusion number)) – 5), which was rounded to the nearest integer. The nose poking requirements were as follows: 1, 2, 4, 6, 9, 12, 15, 20, 25, 32, 40, 50, 62, 77, 95, 118, 145, 178, 219, 268, 328, and 402. After METH self-administration training for 10 days under the FR1 schedule, the rats were randomly divided into five groups (*n* = 7 in each group) and injected (s.c.) with vehicle or buprenorphine at 0.01 mg/kg, 0.03 mg/kg, 0.1 mg/kg, or 0.3 mg/kg at 15 min prior to the training session, when the training procedure was switched to the PR schedule for 4 h. The last successfully completed ratio was registered as the breakpoint for that session (38, 39).

### Drug-seeking induced by context and reinstatement by conditioned cues or methamphetamine priming

After the rats were withdrew for 14 days in their individual housing cages after 14 days METH self-administration, the rats were divided into five groups (n = 7 per group) for receiving vehicle (saline, s.c.), buprenorphine (0.01, 0.03, 0.1, or 0.3 mg/kg, s.c.) to test the drug-seeking behavior induced by context for 2 hours. At 15 min after buprenorphine, rats were re-placed into the same training chambers without house light, LED light, or sound from the pump, and the intake of METH injections by touching the active nose poke. However, the computer recorded the number of active or inactive nose pokes.

The rats underwent 2 h extinction for 3 consecutive days to reduce the effect of context on reinstatement test. The extinction conditions consisted of only original training context, while the pump and lights being turned off. On the reinstatement test day, the rats was performed for 2h in which the rats were exposed to light and noise cues for 5 s at the start of the session, and each subsequent active nose poke previously paired with METH injection elicited a cue presentation without a METH injection for the rest of the test session. The doses of buprenorphine were used as same as described above.

The reinstatement of METH priming was carried out after another 3 consecutive days extinction, rats were administered METH (0.5 mg/kg, i.p.) 10 min before testing, and no conditioned cues were present during the 2-h testing session (36). Rats (n = 7 per group) were injected (s.c.) with buprenorphine at 0.01, 0.03, 0.1 mg/kg, or 0.3 mg/kg or vehicle 15 min prior to methamphetamine (0.5 mg/kg) to test the effect of buprenorphine on drug seeking induced by METH priming.

To elucidate the role of NOP receptor in the effects of buprenorphine on reinstatement of METH priming, four group of rats were injected with vehicle(saline plus 1% DMSO, s.c.), buprenorphine treated group(0.1 mg/kg buprenorphine plus 1% DMSO, s.c.), SB-612111 treated group (1mg/kg SB-612111 plus saline, s.c.), or both SB-612111 (1mg/kg, s.c.) and buprenorphine(0.1mg/kg, s.c.) at 15 min prior to methamphetamine (0.5 mg/kg, i.p.) administration.

### Sucrose reinforcement

The standard procedure stipulated that during one training period, the rat obtained one sucrose pellet every time the nasal touch is correctly completed, and the training period is automatically terminated after 100 pellet deliveries or 1 h. Starting from FR1, when all rats completed 100 sucrose pellets in two training sessions, the FR was increased, and FR2, FR3, FR5, and FR10 sucrose intensive training periods were completed in sequence. Rats underwent one training session per day. After the rats acquired food self-administration under FR10 schedule for 10 days, they were randomly assigned to five groups (*n* = 6 in each group) and injected with vehicle,0.01, 0.03, 0.1, 0.3, and 1 mg/kg buprenorphine (s. c.) at 15 min before the testing session.

### Statistical analysis

Data from the self-administration and reinstatement tests were analyzed by using one-way analysis of variance (ANOVA). Normal distribution and uniform variance were analyzed by Tukey-HSD for *post hoc* analysis between the groups and Games-Howell and LSD multiple comparisons were used for *post hoc* analysis. When the measurement data with uneven variance between the groups and LSD test for pairwise comparison. The mean number of infusions or responses for active and inactive holes during self-administration with FR schedule and the reinstatement by conditional cues and drug priming of METH were analyzed using one-way ANOVA with Tukey-HSD for *post hoc* analysis. The data of breakpoint under PR schedule, thienorphine treatment combined with buprenorphine for METH reinforcement, and sucrose reinforcement were analyzed by one-way ANOVA with Games-Howell. For the data of reinstatement by contextual cues, LSD test was used for pairwise comparison. Statistical significance was considered when the *P*-value was less than 0.05.

## Results

### Effect of buprenorphine on METH reinforcement and motivation

**FIGURE 1:**
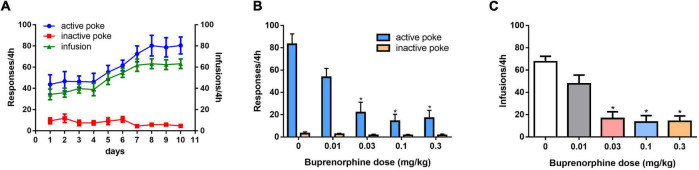
Effects of pretreatment with buprenorphine on methamphetamine reinforcement in rats. Values are presented as means ± SEM. **(A)** The acquisition of METH self-administration at dose of 0.05 mg/kg under FR1 schedule of reinforcement, left *Y* axis indicates the responses of nose poke and the right *Y* axis is the infusions. **(B)**. Buprenorphine inhibited the active responses in a dose-dependent manner on METH self-administration under FR1 schedule. **(C)**. Buprenorphine inhibited infusions on METH self-administration under FR1 schedule. *P < 0.05 vs. vehicle.

As shown in Figure 1A, self-administration of METH was successful after 10 days of training under the FR1 schedule. One-way ANOVA revealed a significant effect of buprenorphine treatment on active nose pokes (*F*_(4, 30)_ = *13.752*, P < 0.001; Figure 1B), but not on inactive nose pokes (*F*_(4, 30)_ = *2.413, P* = *0.071*; Figure 1B). As shown in Figure 1C, one-way ANOVA revealed that T the number of infusions of METH was reduced by buprenorphine treatment at doses ranging from 0.03 to 0.3 mg/kg (*F*_(4, 30)_ = 16.637, *P* < 0.001).

**FIGURE 2:**
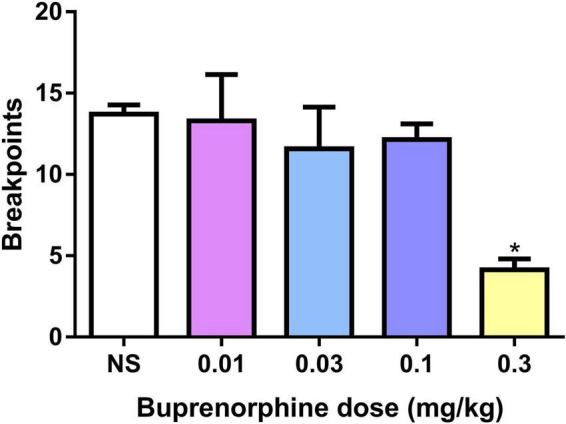
Effects of buprenorphine on the motivation for methamphetamine use. The motivation for methamphetamine use expressed as the breakpoint reached under a progressive-ratio schedule of reinforcement. Values are presented as means ± SEM. *P < 0.05 vs. vehicle.

The effect of buprenorphine on METH motivation was examined under the PR schedule. One-way ANOVA revealed that buprenorphine significantly decreased the breakpoint of active responses (*F*_(4, 30)_ = *4.602, P* = *0.005*; Figure 2A). And at the dose of 0.3 mg/kg, buprenorphine decreased the last number of infusions under the PR schedule (*F*_(4, 30)_ = *3.302, P* = *0.023*; Figure 2B).

### Effect of thienorphine or SB-612111 combined with buprenorphine on methamphetamine reinforcement

**FIGURE 3:**
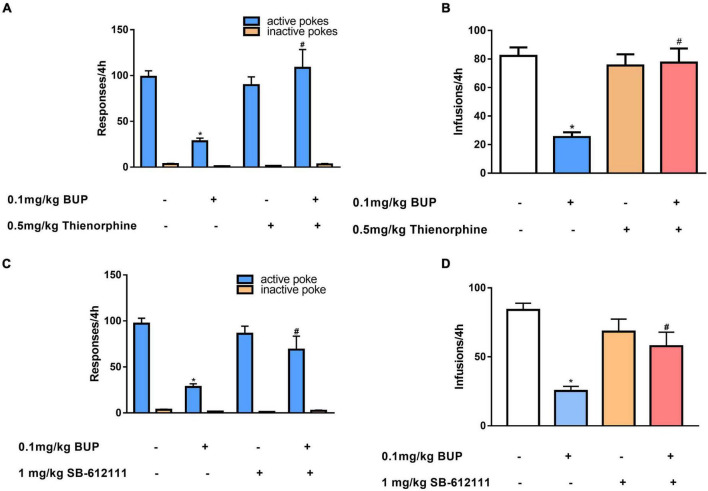
Effects of thienorphine and SB-612111 on inhibitory action of buprenorphine on methamphetamine self-administration. Data are presented as means ± SEM. The effects of thienorphine pretreatment (0.5 mg/kg, s.c.) with buprenorphine on active or inactive response **(A)** and total infusions **(B)** during METH self-administration under FR1 schedule of reinforcement. Buprenorphine reduced the active responses and infusions, thienorphine combined with buprenorphine reversed the inhibitory action of buprenorphine, but thienorphine alone did not affect the responses and infusions. The effects of SB-612111 pretreatment (1mg/kg, s.c.) with buprenorphine on the responses **(C)** and METH infusions **(D)**. SB-612111 reversed the inhibitory action of buprenorphine on the active responses and METH infusions, but it alone failed to affect the responses and infusions. *P < 0.05 vs. vehicle treatment, #*P* < 0.05 vs. buprenorphine alone.

First, we observed the effect of thienorphine treatment on inhibitory action of buprenorphine on METH reinforcement. One-way ANOVA revealed the main effect of active pokes (*F*_(3, 24)_ = 9.776, *P* < 0.001; Figure 3A) and infusions among the four groups (*F*_(3, 24)_ = 13.485, *P* < 0.001; Figure 3B). As shown in Figures 3A,B, 0.1 mg/kg buprenorphine significantly reduced the number of active pokes and METH infusions (*P* < 0.05), but thienorphine alone was not able to reduce the number of active responses (*F* = 4.20, *P* = 0.943) and infusions (*F* = 4.295, *P* = 0.976). When thienorphine and buprenorphine were co-administered, the number of active responses and infusions increased significantly compared to buprenorphine alone (both *P* < 0.05). Inactive responses did not differ among the four groups (F_(3, 24)_ = 2.955, *P* = 0.053).

Next, we determined the effects of another NOP antagonist SB-612111 on inhibitory action of buprenorphine on METH reinforcement. One-way ANOVA revealed a significant main effect of active pokes (F_(3, 24)_ = 11.081, *P* < 0.001, Figure 3C) and infusions (F_(3, 24)_ = 11.105, *P* < 0.001; Figure 3D). Multiple comparisons showed that active pokes and infusions pretreated by buprenorphine decreased significantly compared with the vehicle (*P* < 0.05). However, no significant effect of SB-612111 alone on active pokes (*F* = 0.87, *P* = 0.827) or infusions (*F* = 2.51, *P* = 0.458) was observed. When SB-612111 and buprenorphine co-administered, the active pokes (*F* = 8.182, *P* = 0.009) and infusions (*F* = 10.438, *P* = 0.004) were significantly increased compared with those in the buprenorphine alone. There were no differences in the inactive responses among the four groups (F_(3, 24)_ = 2.980, *P* = 0.051; Figure 3C).

### Effect of buprenorphine on drug-seeking induced by context and reinstatement of conditioned cues

**FIGURE 4:**
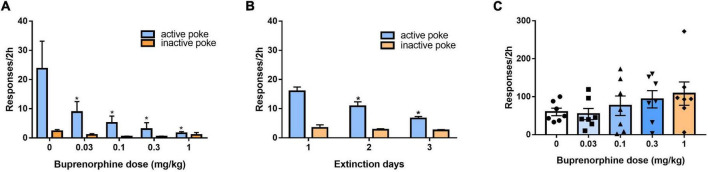
Effect of buprenorphine on drug-seeking induced by cues. **(A)** Buprenorphine pretreatment reduced the active responses induced by context during 2 h test in the training chamber after withdrawal from METH self-administration for 14 days. *P < 0.05 vs. vehicle treatment. **(B)** The rats were extinguished for 3 days. *P < 0.05 vs. first day extinction. **(C)** The reinstatement induced by conditioned cues. Buprenorphine did not inhibit the active responses induced by conditional stimulus cues during 2 h test. Data are presented as means ± S.E.M.

We evaluated the effect of buprenorphine on context-induced drug-seeking behavior after withdrawal for 14 days. As shown in Figure 4A, one-way ANOVA revealed significant effect of buprenorphine on the active nose pokes (*F*_(4, 30)_ = 3.559, *P* = 0.017), the multiple comparison showed that the active responses were reduced by buprenorphine at the doses of 0.1,0.3 or 1 mg/kg (all *P* < 0.05), while the inactive nose pokes were not significantly different among the groups (*F*_(4, 30)_ = 2.203, *P* = 0.093). This indicated that buprenorphine inhibited in a dose dependent manner drug-seeking behavior induced by contextual cue.

After 3 days of extinction, the rats were tested to evaluate the effects of reinstatement of METH seeking induced by conditioned cues. As shown in Figure 4B, one-way ANOVA revealed significant effect of extinction (days) on the active responses (*F*_(2, 102)_ = 12.806, *P* < 0.001) but no effect on the inactive responses (*F*_(2, 102)_ = 0.518, *P* = 0.597). As shown in Figure 4C, buprenorphine tended to increase the active responses, but one-way ANOVA revealed no significant main effect of buprenorphine on the active responses (*F*_(4, 30)_ = 1.048, *P* = 0.399) or inactive nose pokes (*F*_(4, 30)_ = 2.665, *P* = 0.052).

### Effects of SB-612111 combined with buprenorphine on reinstatement of methamphetamine priming

**FIGURE 5:**
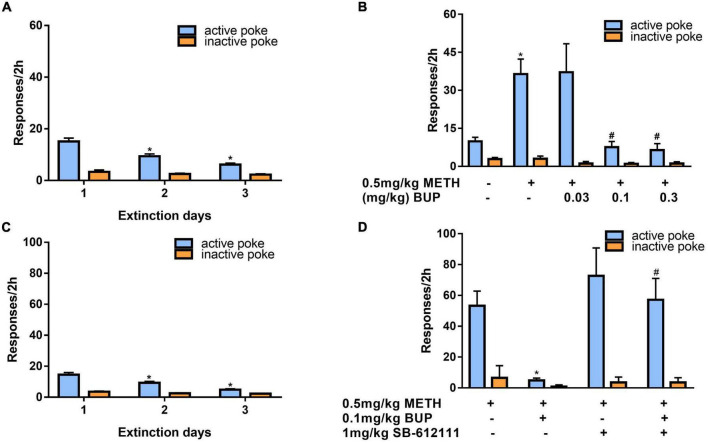
Effects of buprenorphine or SB-612111 combined with buprenorphine on drug-seeking induced by METH priming in rats. **(A)** The rats were extinguished for 3 days. *P < 0.05 vs. first day extinction. **(B)** Effects of buprenorphine on drug-seeking behavior induced by METH priming. The active responses increased significantly after administration of METH and buprenorphine inhibited the active responses in a dose-dependent manner. *P < 0.05 vs. vehicle, #*P* < 0.05 vs. buprenorphine alone. **(C)** The rats were extinguished for 3 days. *P < 0.05 vs. first day extinction. **(D)** Effects of SB-612111 combined with buprenorphine on drug-seeking behavior induced by METH priming. SB-612111 pretreatment reversed the inhibitory action of buprenorphine on active responses induced by METH priming, but it alone failed to affect the active responses. Data shown are means ± S.E.M. *P < 0.05 vs. METH priming, #P < 0.05 vs. buprenorphine treatment.

After 3 days of additional extinction (Figure 5A), the rats were tested to observe the effects of buprenorphine on reinstatement of METH priming. One-way ANOVA revealed significant effect of extinction (days) on the active responses (*F*_(2, 102)_ = 21.617, *P* < 0.001) but no effect on the inactive responses(*F*_(2, 102)_ = 0.984, *P* = 0.377). As shown in Figure 5B, one-way ANOVA revealed a significant main effect of buprenorphine on the active nose pokes (*F*_(4, 30)_ = 7.134, *P* < 0.001) but not the inactive nose pokes (*F*_(4, 30)_ = 1.710, *P* = 0.714). Multiple comparisons indicated that METH administration could significantly increase the active responses compared to that of vehicle group (*P* < 0.05), indicating that METH priming induces the reinstatement of drug seeking behavior. Additionally, buprenorphine at the doses of 0.3 to 1 mg/kg significantly decreased the active responses compared to that of METH primed group (*P* < 0.05).

After another 3 days of extinction (Figure 5C), the rats were tested to observe the effects of SB-612111 combined with buprenorphine on reinstatement of METH priming. One-way ANOVA revealed significant effect of extinction (days) on the active responses (*F*_(2, 81)_ = 23.161, *P* < 0.001) but no effect on the inactive responses (*F*_(2, 81)_ = 4.045, *P* = 0.105). As shown in Figure 5D, one-way ANOVA revealed the main effect of SB-612111 combined with buprenorphine on the active nose pokes (*F*_(3, 21)_ = 5.627, *P* = 0.005), whereas the inactive nose pokes were not significantly different among four groups (*F*_(3, 21)_ = 1.802, *P* = 0.714). Multiple comparisons indicated that buprenorphine at 0.1 mg/kg significantly decreased the active responses compared with the vehicle (*F* = 6.700, *P* = 0.049) and the combination administration of SB-612111 with buprenorphine increased the active responses compared with buprenorphine alone (*F* = 8.413, *P* = 0.03). However, SB-612111 alone failed to affect the active responses (*F* = 1.766, *P* = 0.686).

### Effect of buprenorphine on sucrose reinforcement

**FIGURE 6:**
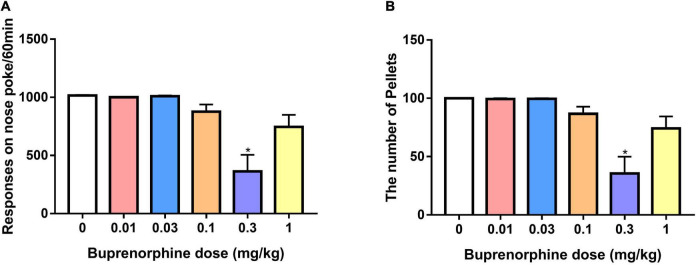
Effect of buprenorphine on sucrose self-administration. Data are presented as means ± SEM. **(A)** The effect of buprenorphine on the responses of nose pokes under FR10 schedule. **(B)** The effect of buprenorphine on the number of sucrose pellets. Only buprenorphine at dose of 0.3 mg/kg inhibited the responses and sucrose pellets. *P < 0.05 vs. vehicle.

To determine whether buprenorphine specifically affected METH reinforcement, the effect of buprenorphine on sucrose self-administration was examined in a separate set of rats. As shown in Figure 6, one-way ANOVA revealed a significant effect of buprenorphine on the responses (*F*_(5, 36)_ = 10.999, *P* < 0.001) and sucrose pellets (*F*_(5, 36)_ = 10.793, *P* < 0.001). Multiple comparisons indicated that buprenorphine significantly reduced the responses and total number of sucrose pellets only at the doses of 0.3 mg/kg (both *P* < 0.05).

## Discussions

The present findings showed that buprenorphine pretreatment reduced the rewarding effect, total consumption, and rewarding motivation of METH, and this effect was reversed by the NOP receptor antagonists thienorphine and SB-612111. Moreover, buprenorphine inhibited the drug-seeking behavior induced by context or METH priming but failed to reduce the drug-seeking behavior induced by conditioned cues. The inhibitory action of buprenorphine on METH priming-induced drug-seeking behavior was reversed by the NOP receptor antagonist SB-612111. These results demonstrated that buprenorphine not only attenuated the METH self-administration but also the relapse into drug-seeking behavior through the activation of NOP receptor.

Buprenorphine is widely used to treat opioid addiction (40) and also blocks the action of exogenous opioids, thereby reducing the use of illegal opioids (41). The present results showed that low-dose buprenorphine treatment inhibited METH self-administration and total intake doses of METH in rats. Evaluating the dose effects of buprenorphine on food rewards indicated that small doses of buprenorphine were unlikely to inhibit natural rewards. Moreover, the evidence have shown that BUP at 0.1 mg/kg significantly increases locomotor activity compared to vehicle controls (42), METH and buprenorphine has no effects on locomotor activity in the open field test (43). This suggested that buprenorphine at lower doses may have a specific inhibitory effect on METH reinforcement and consumption. Albeit buprenorphine reduced incentive motivation for METH at 0.3 mg/kg, this dose of buprenorphine also inhibited the sucrose reinforcement, indicating no specific effect of buprenorphine on motivation for METH.

Under normal circumstances, DA is released and the DA transporter (DAT) on the presynaptic membrane can reuptake DA to maintain it at a stable concentration in the synaptic cleft (44). METH, as a pseudo-neurotransmitter, can bind with DAT, resulting in the uncontrolled release of DA in the NAc (45). As the reuptake of DA is inhibited, the DA content in the synaptic cleft sharply increases, with the eventual exhaustion of DA during long-term METH exposure (46). Buprenorphine is a partial agonist of MOP, an antagonist of DOP and KOP (47), and a low-affinity partial agonist of the NOP receptor (19, 48). Studies have shown that MOR agonists can modulate the activity of dopamine neurons, thus altering the pharmacodynamic effects of METH on the dopaminergic system (49). Buprenorphine attenuates the METH-induced DA peak effect, and at low doses, it reduces METH-induced DA release (18). Buprenorphine prevents acute novelty stress-induced blunting of DA levels and approach behavior for food reward (50). However, buprenorphine activates DA neurons in the VTA, but this activation is not reversed by the opioid antagonist naloxone (51). Buprenorphine also enhances basal levels of DA, attenuates the NAc DA response to heroin, and enhances the DA response to cocaine (15). Although blockade of classical MOP by naltrexone is not sufficient to prevent METH self-administration (52). Recent evidence has demonstrated that co-activation of NOP and MOP receptors is essential for buprenorphine to reduce cocaine intake (14). Through coactivation of NOP and MOP receptors, bifunctional NOP/MOP receptor agonists can attenuate opioids and other abused drugs (53).Thus, buprenorphine regulates METH consumption through its unique and complex pharmacological effects.

N/OFQ and its NOP receptors expressed in the medial prefrontal cortex, VTA, and NAc exert a number of functional effects, including blocking stress-induced analgesia, anxiolytic-like effects, and reducing drug rewards (54). Accordingly, N/OFQ mRNA is expressed largely on GABA neurons, whereas NOP receptor mRNA is located on DA neurons. N/OFQ is in a position to influence DA neuronal activity by means of the NOP located on DA neurons (55). Moreover, intraventricular injection with N/OFQ or NOP receptor agonists significantly reduces alcohol intake and alcohol self-administration (56). N/OFQ blocks cocaine CPP (26) and maladaptive behavioral changes induced by repeated cocaine treatment (25) or rewarding properties of morphine and psychostimulants (27, 41). Buprenorphine has dual effects as an opioid receptor ligand; higher doses reduce ethanol consumption via the activation of NOR receptors (20). To elucidate this inhibitory mechanism, we performed pharmacological studies using the NOP antagonists thienorphine and SB-612111. SB-612111 behaves *in vivo* as a potent and selective NOP antagonist (32). Thienorphine, a novel analog of buprenorphine, can bind NOP but results in inactive stimulation, thereby antagonizing NOP (31). In the present study, neither thienorphine nor SB-612111 alone changed METH self-administration, indicating that the endogenous NOP system was not involved in the METH reinforcement behavior. However, their combined treatment with buprenorphine reversed the inhibition of METH reinforcement by buprenorphine, suggesting that the inhibition of METH reinforcement by buprenorphine may be mediated mainly through the activation of NOP receptor.

Buprenorphine treatment inhibited context or METH priming-induced METH-seeking behavior. However, it failed to affect the conditioned cues induced drug-seeking behavior. These results are similar to those of a previous report that buprenorphine reduces cocaine-seeking during extinction and following acute cocaine priming injections, but has no effect on stress-induced reinstatement (16). The exact mechanism by which buprenorphine modulates context or drug priming-induced drug-seeking behavior is not yet clear. First, the different circuits and mechanisms underlying relapse induced by contextual cues, conditioned cues, or drug priming are considered (57, 58). For example, a series of projections, primarily involving dopamine from the VTA to the NAc shell and glutamate from the BLA or dmPFC to the NAc core, appear to be the primary pathways mediating conditioned cue-induced reinstatement (59). The dmPFC projections to the NAc core and dopamine innervations of the vmPFC and NAc shell are likely involved in drug-primed reinstatement (60). The dorsal hippocampus and NAc shell play a significant role in the contextual reinstatement of drug seeking (61). The contextual cue-induced heroin relapse behavior may be the result of involvement of the hippocampal-NAc glutamate pathway and the VTA-NAc DA pathway (62). Buprenorphine enhances basal levels of DA (15) and increased basal levels of glutamate in drug-naïve and cocaine-exposed rats (17), which may facilitate CS salience. This possibility is further supported by data showing that naltrexone reduces the reinstatement of drug seeking induced by METH-associated cues (52, 63). Thus, the discrepant effects of buprenorphine on drug-seeking behavior induced by contextual cues and conditional cues may be related to the different mechanisms.

Another explanation is that buprenorphine may activate NOP to reduce DA release and inhibit contextual cue or drug priming-induced seeking behavior. Thus, N/OFQ administration prevents the reinstatement of ethanol-seeking behavior elicited by contextual cues (56). The present results showed that a NOP antagonist could reverse the inhibitory action of buprenorphine on METH priming drug-seeking behavior, which is consistent with previous reports. For example, genetic deletion of NOP receptors decreases heroin, cocaine, or alcohol self-administration and CPP (64) and potent and selective activation of NOP receptors is sufficient to decrease cocaine intake and seeking behavior in rats (65). These findings support the notion that low-dose buprenorphine is a weak dopamine releaser relative to heroin and METH, and that buprenorphine pretreatment can block the dopamine-releasing effects of heroin and METH (66).

Opioid receptor agonists can modulate the activity of dopamine neurons and can therefore modify the pharmacodynamic effects of METH on the dopaminergic system. The efficacy of adjunctive medication with buprenorphine has been demonstrated in the treatment of cocaine addiction, extending beyond opiate addiction. A few clinical trials have shown that buprenorphine maintenance decreases craving for METH in METH users (11, 12). Based on the efficacy of buprenorphine on heroin dependence, this study offers supporting evidence that buprenorphine may be used for the treatment of METH dependence. We systematically observed and analyzed the effects of buprenorphine on METH intake and relapse behaviors and found that buprenorphine has an inhibitory effect on METH self-administration, reward motivation, and drug-seeking behavior induced by drug priming. Meanwhile, it is cautious to clinical trials of buprenorphine for METH use disorder because buprenorphine may slightly stimulate the drug seeking induced by cues. Interestingly, naltrexone reduces the reinstatement of drug seeking induced by conditioned cues, on the other hand, it fails to affect the reinstatement induced by METH-priming (52, 63). Moreover, low doses of risperidone also can inhibit the drug seeking induced by conditioned cues (67, 68).Thus, it will be beneficial to use buprenorphine in conjunction with other medicines such as naltrexone or risperidone to block the drug-seeking behavior induced by cues and drug priming.

Taken together, our results demonstrated that buprenorphine has a significant inhibitory effect on key aspects of METH dependence. Therefore, the present results suggested that buprenorphine can be used as an adjunctive therapy for the METH use disorders and relapse prevention.

## Data availability statement

The original contributions presented in this study are included in the article/supplementary material, further inquiries can be directed to the corresponding author/s.

## Ethics statement

The animal study was reviewed and approved by Ethics Committee of the Laboratory Animal Use and Care of Ningbo University.

## Author contributions

FW, WS, and YC performed the experiments, analyzed the data, and wrote the manuscript. XZ, HD, and ML performed the experiments. HL was responsible for the study concept and supervised the experiments. EK and WZ was responsible for study design and critically revised the manuscript. All authors critically reviewed content and approved final version for publication.
